# A Matching-Adjusted Indirect Comparison of Sonidegib and Vismodegib in Advanced Basal Cell Carcinoma

**DOI:** 10.1155/2017/6121760

**Published:** 2017-05-21

**Authors:** Dawn Odom, Deirdre Mladsi, Molly Purser, James A. Kaye, Eirini Palaka, Alina Charter, Jo Annah Jensen, Dalila Sellami

**Affiliations:** ^1^RTI Health Solutions, 200 Park Offices Dr., Research Triangle Park, NC, USA; ^2^Novartis Pharma AG, Forum 1, Novartis Campus, 4056 Basel, Switzerland; ^3^Novartis Pharmaceuticals Corporation, One Health Plaza, East Hanover, NJ, USA

## Abstract

**Objectives:**

Based on single-arm trial data (BOLT), sonidegib was approved in the US and EU to treat locally advanced basal cell carcinomas (BCCs) ineligible for curative surgery or radiotherapy. Vismodegib, the other approved targeted therapy, also was assessed in a single-arm trial (ERIVANCE). We examined the comparative effectiveness of the two drugs using a matching-adjusted indirect comparison (MAIC) versus an unadjusted indirect comparison.

**Methods:**

After comparing trials and identifying potential prognostic factors, an MAIC was conducted to adjust for differences in key patient baseline characteristics. Due to BOLT's small sample size, the number of matching variables was restricted to two. Efficacy results for sonidegib were generated so that selected baseline characteristics matched those from ERIVANCE and were compared with published ERIVANCE results.

**Results:**

Matching variables were baseline percentages of patients receiving prior radiotherapy and surgery. After weighting, sonidegib objective response rate (ORR) and median progression-free survival (PFS) were effectively unchanged (prematched versus postmatched ORR and PFS, 56.1% versus 56.7% and 22.1 versus 22.1 months, resp.). Vismodegib's ORR and PFS were 47.6% and 9.5 months.

**Conclusions:**

Comparative effectiveness of sonidegib versus vismodegib remains unchanged after adjusting BOLT patient-level data to match published ERIVANCE baseline percentages of patients receiving prior surgery and radiotherapy.

## 1. Introduction

Basal cell carcinoma (BCC) is one of the most prevalent cancers [1] and generally is diagnosed and treated early [2]. However, in some cases, BCC metastasizes or progresses locally to the extent that curative surgery or radiotherapy is not feasible [3]. Advanced BCC can cause disfigurement and morbidity and lower patients' quality of life [4, 5]. Two oral treatments that target the hedgehog pathway have become available recently for patients with advanced BCC: sonidegib (Odomzo; Novartis) and vismodegib (Erivedge; Roche). Based on results from a noncomparative study (the 200 mg arm of the BOLT trial) [5], sonidegib was approved recently in the United States and European Union to treat adults with locally advanced BCC (laBCC) who are ineligible for curative surgery or radiotherapy [6, 7]. Similarly, vismodegib, the other approved targeted oral therapy for advanced BCC, was approved based on a single-arm trial (ERIVANCE) [8]. Vismodegib is indicated for the treatment of adults with symptomatic metastatic BCC or adults with laBCC who are ineligible for surgery or radiotherapy [9].

No study to date has directly compared the efficacy of these two hedgehog pathway inhibitors, and no studies have been reported which evaluate these drugs versus a common comparator. Yet clinicians may be interested in how the efficacies of vismodegib and sonidegib compare in patients with laBCC who are ineligible for surgery or radiotherapy [10]. Payers also may need comparative effectiveness information to facilitate cost-effectiveness assessment. In an unadjusted (“naive”) indirect comparison of the two treatments, point estimates from BOLT show a longer median progression-free survival (PFS) and a higher objective response rate (ORR) than those observed in ERIVANCE. However, patients in the two trials had different distributions of potentially prognostic baseline characteristics, which might confound the treatment comparison.

In the absence of a direct treatment comparison in a randomized trial of sonidegib versus vismodegib or separate randomized trials versus a common comparator, we conducted a matching-adjusted indirect comparison (MAIC). The MAIC method is designed to reduce confounding of treatment effects by aligning the distributions of important patient characteristics between, for example, two single-arm trial populations. In this case, individual patient data (IPD) from one trial are weighted to match mean baseline characteristics as published from the second trial [11, 12]. Results of the trial with IPD are then reanalyzed using the weighted patient-level data set. MAIC increasingly has been used in health technology assessment submissions in the past few years [13]. When implemented correctly and transparently, it can provide an alternative for comparative effectiveness data in the absence of head-to-head randomized trials [13]. The objective of this analysis was to assess the comparative effectiveness of sonidegib and vismodegib, based on two single arms of noncomparative trials, in patients with laBCC who are ineligible for curative surgery or radiotherapy, adjusting for differences in selected patient baseline characteristics using an MAIC versus making an unadjusted indirect comparison.

## 2. Materials and Methods

Before performing the MAIC, we conducted a critical review and comparison of the BOLT and ERIVANCE trial designs, outcome definitions, and baseline patient characteristics to determine the suitability for an indirect comparison of sonidegib and vismodegib. A targeted literature review was undertaken and augmented with consultation from clinical advisors to identify baseline patient characteristics that may be considered prognostic for the outcomes of interest. After the baseline characteristics to be used in the MAIC procedure were determined, the MAIC was conducted and the results were compared with those from an unadjusted indirect comparison.

### 2.1. Assessment of Trials and Selection of Analysis Parameters

The BOLT and ERIVANCE trials [5, 8] were assessed to determine suitability for conducting an indirect comparison of sonidegib and vismodegib for the treatment of patients with laBCC who are ineligible for curative surgery or radiotherapy. From BOLT, only the laBCC subpopulation in the sonidegib 200 mg arm was included in this analysis; the other subpopulation (metastatic BCC) and dose (800 mg) were not included in the product label. In general, the trial designs and eligibility criteria were similar. As shown in Table 1, both studies were multicenter, international clinical trials for patients with histologically confirmed diagnoses (measurable disease of ≥1 lesion, ≥10 mm in at least one dimension) and not amenable to surgery. The endpoints of the trials were defined similarly, with the exception of response, which was based on more stringent criteria in BOLT. There were a few differences in the exclusion criteria between the trials, including requirements for prior radiotherapy, life expectancy, and presence of superficial multifocal BCC that may be considered unresectable due to breadth of involvement.

The BOLT 18-month update (18 months following enrollment of the last patient) and the ERIVANCE 12-month update (12 months of follow-up after the 9-month study, resulting in 21 months following enrollment of the last patient) were considered to be closely aligned (see Table 1) and provided the longest common duration of follow-up at the time of the analysis; therefore, these data cutoff times were selected for the analysis.

The two clinical trials evaluated several similar efficacy endpoints. ORR was selected as an outcome to be analyzed because it was the primary efficacy endpoint in both trials. PFS was selected as an additional outcome for analysis because PFS can facilitate development of a cost-effectiveness model according to best practices in advanced cancer, which is important for payers and health technology assessment authorities. Duration of response (DOR) was selected as an additional outcome because it may be clinically relevant in this patient population.

For the efficacy outcomes, Response Evaluation Criteria in Solid Tumors (RECIST) were used to assess tumor response in both studies. However, BOLT used modified RECIST (mRECIST) criteria, which defined response based on composite evidence from magnetic resonance imaging, color photography, and histology. mRECIST is a more stringent tool for evaluating tumor response compared to the response criteria used in ERIVANCE [23, 24]. To assess the effect of the more stringent response criteria on the ORR and DOR observed in BOLT, a sensitivity analysis was conducted which applied, to the BOLT IPD, response criteria that more closely aligned to the response criteria used in ERIVANCE.

Table 2 summarizes the potential prognostic characteristics that were available for MAIC matching. Given the small sample size of BOLT (*n* = 66 for laBCC, 200 mg), there was concern that including all reported baseline variables in the matching procedure would lead to extreme weights and unstable results. Therefore, the number of matching variables was limited to two, which were selected a priori based on the following criteria: (1) available and presented consistently in both BOLT and ERIVANCE, (2) distributed differently between BOLT and ERIVANCE (based on visual review), and (3) prognostic for the efficacy outcomes (based on input from clinical advisors and supplemented by literature as necessary). Prior BCC radiotherapy and prior BCC surgery were selected as the two matching variables for MAIC (Table 3).

### 2.2. Statistical Methods

All analyses were conducted using SAS statistical software version 9.3 or higher. A naive indirect comparison based on unadjusted results from both studies was conducted as a base case. The statistical methodology detailed by Signorovitch et al. [11] and Signorovitch et al. [12] was implemented for the MAIC analysis. Using this method, patients in the sonidegib study (i.e., for whom IPD data were available) were weighted so that their selected baseline characteristics (proportions) matched the selected aggregate baseline characteristics reported for the published vismodegib study. The Newton-Raphson algorithm was used to obtain the unique solution for the weights (using SAS NLPNRA subroutine and GRD option in PROC IML). Because the small sample size in the trials was a concern, weights were examined for extreme values. After matching, weighted statistical analysis of the key sonidegib study efficacy endpoints was produced. Specifically, a weighted statistical analysis of the sonidegib IPD was applied using SAS via a weighted chi-square test (PROC FREQ) or weighted Kaplan-Meier analysis (PROC LIFETEST). Finally, the two treatments were compared in a way similar to a naive indirect comparison but using the reweighted sonidegib results.

## 3. Results and Discussion

### 3.1. Results

The analysis includes 66 patients with laBCC in the BOLT sonidegib 200 mg arm and published results based on 63 patients in ERIVANCE (vismodegib 150 mg). Patient baseline characteristics are summarized in Table 4. A higher percentage of patients in the vismodegib study received prior radiotherapy for BCC compared to those in the sonidegib study. The percentage of patients who had received prior surgery for BCC was also higher in the vismodegib study. After matching on these two characteristics, the percentages of sonidegib patients were similar to the percentages of vismodegib patients in these baseline characteristics (Table 4). The calculated weights had a mean equal to 1 (standard deviation, 0.573; range, 0.40–2.72). The unmatched patient characteristics of age, age range, race, Eastern Cooperative Oncology Group (ECOG) status, sex, and prior systemic therapy were comparable before and after matching (Table 4).

Table 5 presents the unadjusted (i.e., prematched) results for sonidegib, the MAIC-adjusted (i.e., postmatched) results for sonidegib, and the published results for vismodegib. In the unadjusted comparison between sonidegib and vismodegib, the ORR estimate (95% confidence interval) for sonidegib was 56.1% (44.1%–68.0%), slightly higher than the published ORR estimate for vismodegib of 47.6% (35.5%–60.6%), with the 95% confidence intervals overlapping. Median PFS estimates were longer for sonidegib at 22.1 months (14.8, not estimable) compared with vismodegib at 9.5 months (7.4–14.8), with nearly separated confidence intervals. Median DOR based on investigator review was 14.3 months (12.0–20.2) for sonidegib and was not reached for vismodegib at the time of analysis. Median DOR based on independent review was reported to be 9.5 (7.4–21.4) months for vismodegib. Because the median DOR based on investigator review was not reached for vismodegib, a comparison between the treatments could not be made.

After applying the matching adjustment, the ORR estimate for sonidegib changed slightly from the naive result [(56.1% [44.1–68.0]) to (56.7% [44.7–68.6])] and median PFS was unchanged (22.1 months [14.8, not estimable]) (Table 5). For DOR based on investigator review, the median point estimate for sonidegib increased slightly to 15.7 months (12.9–23.1).

In the sensitivity analysis applying the ERIVANCE-like criteria to assess tumor response to the weighted IPD from BOLT, pre- and postmatched ORRs for sonidegib were 60.6% (48.4–72.4) and 59.5% (47.6–71.3), respectively. Median DORs before and after matching for sonidegib were 14.9 (12.0–20.2) and 15.7 (12.9–24.0), respectively.

### 3.2. Discussion

In the absence of a randomized trial directly comparing sonidegib versus vismodegib or even separate randomized trials using a common comparator (e.g., placebo), a naive indirect treatment comparison is the only simple way to gauge the relative efficacy of these two targeted therapies approved in locally advanced BCC. Because naive-treatment comparisons are subject to selection bias, we conducted a MAIC to reduce potential confounding of the treatment effects by aligning the distributions of important patient characteristics, in our case prior BCC radiotherapy and prior BCC surgery. The results of the MAIC we conducted did not change the overall conclusions from the unadjusted naive indirect comparison of the two trials in that patients in the sonidegib study had a slightly higher ORR, longer median PFS, and potentially longer median DOR compared to those in the vismodegib trial prior to the MAIC and the postmatched findings were similar. Although the confidence intervals around the results for these endpoints were not clearly separated from one another (comparing sonidegib versus vismodegib for each endpoint), the magnitude of the observed difference in the point estimates for PFS (more than twofold, favoring sonidegib) is a potentially clinically important result.

The matching procedure was effective in that the postmatched BOLT laBCC population had similar proportions of patients who had received prior BCC radiotherapy and prior BCC surgery compared with the ERIVANCE laBCC population. To the extent that these characteristics are associated with a difference in prognosis, improving the balance in their distributions between the two study populations will reduce potential confounding of the outcomes in the indirect comparison. Although the literature on clinical predictors of efficacy in laBCC is sparse, we based our variable selection on the available research supplemented by input from clinical advisors. The matching-adjusted BOLT patient weights were not viewed as extreme, and the small changes from the unmatched baseline variables in other (unmatched) baseline patient characteristics further strengthen confidence in the validity of the MAIC results. In addition, the matching variables were selected prior to conducting the analysis to avoid any potential bias.

MAIC adjusts treatment comparisons for selected baseline characteristics, but no statistical procedure can adjust for unavailable or unknown confounding variables; unobserved differences between the study populations may still result in residual confounding. Moreover, the indirect comparison is not anchored to a common comparator (because the available data are based on two single arms of noncomparative trials), and therefore relative effects (e.g., relative risks and hazard ratios) cannot be examined. The small size of the BOLT 200 mg laBCC patient group could result in the MAIC relying on extreme weights for some matching variables; to address this, a limited number of matching variables were selected, with no extreme weights observed.

Although BCC is a common malignancy, advanced BCC that is difficult to treat with surgery or radiotherapy is much less common [26]. The literature in this population is quite limited and few treatments exist for patients with advanced BCC ineligible for curative surgery or radiotherapy [3]. Recently, developments in laBCC therapies have focused on oral hedgehog inhibitors such as the recently approved vismodegib and sonidegib. These therapies are a promising addition to the limited treatment options in laBCC. Our research attempts to extend the literature by providing researchers and clinicians an understanding of the comparative effectiveness of these two newly approved oral therapies in laBCC.

## 4. Conclusions

In the unadjusted indirect comparison between sonidegib and vismodegib, sonidegib had slightly higher ORR, longer median PFS duration, and potentially longer median DOR compared to vismodegib. Using the MAIC technique, the comparative effectiveness of these two treatments remains unchanged (in relation to a naive comparison) after adjusting BOLT patient-level data to match published ERIVANCE values for baseline prevalence of prior surgery and radiotherapy. Results from the MAIC confirm and provide support for the validity of the naive indirect comparison results.

## Figures and Tables

**Table 1:** Overview of trial designs, including outcome definitions.

Trial characteristics	BOLT	ERIVANCE
Study description	(i) Multicenter, international, randomized, double-blind, phase 2 study to investigate the safety and efficacy of sonidegib (ii) Patients were randomized to receive either 200 mg or 800 mg^a^ of sonidegib [5]	(i) Single-arm, multicenter, international, nonrandomized, phase 2 study to investigate the safety and efficacy of vismodegib [8]

Key inclusion criteria	(i) Histologically confirmed diagnosis, with measurable disease of ≥1 lesion, ≥10 mm in at least 1 dimension by MRI or color photograph (ii) Patients were not amenable to radiation therapy, curative surgery, or other local therapies (iii) Patients were not required to have received any prior therapy [5]	(i) Histologically confirmed diagnosis, with measurable disease of ≥1 lesion, ≥10 mm in the longest dimension (ii) Patients were considered to be inoperable or medically contraindicated to surgery (iii) Patients were required to have been given radiotherapy unless radiotherapy was contraindicated or inappropriate [8]

Key exclusion criteria	(i) Life expectancy was not mentioned (ii) Presence of superficial multifocal BCC that may be considered unresectable was not mentioned [5]	(i) Patients with life expectancy <12 weeks [8] (ii) Patients with superficial multifocal BCC that may be considered unresectable due to breadth of involvement [9]

Periods for reported results (minimum duration of follow-up)	(i) Primary analysis (6 months of follow-up) [14] (ii) 12-Month update (12 months of follow-up) [5] (iii) 18-Month update (18 months of follow-up) [7]	(i) Primary analysis (9 months of follow-up) [8] (ii) 6-Month update (15 months of follow-up) [15] (iii) 12-Month update (21 months of follow-up) [16] (iv) 18-Month update (27 months of follow-up) [17] (v) 24-Month update (33 months of follow-up) [18] (vi) 30-Month update (39 months of follow-up) [19]

Primary efficacy endpoint	(i) ORR by central review	(i) ORR by central review

Other efficacy and safety outcomes available	(i) DOR (ii) Complete response rate (iii) PFS (iv) Overall survival (v) Time to response (vi) Specific adverse events	(i) DOR (ii) Complete response rate (iii) PFS (iv) Overall survival (v) Specific adverse events

Assessment of tumor response	(i) mRECIST: composite assessment of MRI (per RECIST v1.1) [20], photograph (per WHO [21]), and histology (ii) Prespecified sensitivity analysis using ERIVANCE-like criteria	(i) Composite assessment of MRI or photograph (per RECIST v1.0) [22], ulceration, and histology

BCC: basal cell carcinoma; DOR: duration of response; mRECIST: modified RECIST; MRI: magnetic resonance imaging; ORR: objective response rate; PFS: progression-free survival; RECIST: Response Evaluation Criteria in Solid Tumors; WHO: World Health Organization. ^a^Only patients with locally advanced BCC in the 200 mg arm are included in this analysis.

**Table 2:** Overview of trial patient baseline characteristics.

Potential matching variable	Available and presented consistently in BOLT and ERIVANCE?	Distribution differs between BOLT and ERIVANCE?	Is the variable prognostic?
Age	Yes	No (i) BOLT = 64.6 (mean) (ii) ERIVANCE = 61.4 (mean)	BOLT exploratory analysis suggests prognostic [7], but Chang et al. [25] suggest nonsignificant relationship with ORR

Sex	Yes	No (i) BOLT = 57.6% (male) (ii) ERIVANCE = 55.6% (male)	Unknown

Race	Yes	No (i) BOLT = 89.4% white, 10.6% other (ii) ERIVANCE = 100% white	Unknown

ECOG status	Yes	No (i) BOLT: (a) ECOG status 0 = 66.7% (b) ECOG status 1 = 24.2% (c) ECOG status 2 = 6.1% (ii) ERIVANCE (a) ECOG status 0 = 76.2% (b) ECOG status 1 = 20.6% (c) ECOG status 2 = 3.2%	BOLT exploratory analysis suggests prognostic [7]

Prior radiotherapy for BCC	Yes	Yes (i) BOLT = 7.6% (ii) ERIVANCE = 20.6% for target and 27.0% for current or prior	Chang et al. [25] suggest nonsignificant relationship with ORR

Prior systemic therapy for BCC	Yes	No (i) BOLT = 6.1% (ii) ERIVANCE = 11.1% (systematic or topical)	Chang et al. [25] suggest significant relationship with ORR

Prior surgery for BCC	Yes	Yes (i) BOLT = 72.7% (ii) ERIVANCE = 88.9%	Clinical advisors suggest highly prognostic in refractory population

BCC: basal cell carcinoma; ECOG: Eastern Cooperative Oncology Group; ORR: objective response rate. *Note*. BOLT summaries are based on the 200 mg full analysis set population [7]; ERIVANCE summaries are based on Sekulic et al. [8] and the European Medicines Agency assessment report [9].

**Table 3:** Parameters selected for analysis.

Parameter	Selected for analyses
Period of reported results (minimum follow-up period)	(i) BOLT: 18-month update (18 months of follow-up) (ii) ERIVANCE: 12-month update (21 months of follow-up)

Efficacy outcomes	(i) ORR (ii) DOR (iii) PFS

Matching variables	(i) Prior BCC radiotherapy (ii) Prior BCC surgery

BCC: basal cell carcinoma; DOR: duration of response; ORR: objective response rate; PFS: progression-free survival.

**Table 4:** Baseline characteristics.

	BOLT^a^, sonidegib 200 mg		ERIVANCE^a^, vismodegib 150 mg (*n* = 63)
	Prematched (*n* = 66)	Postmatched (*n* = 66)	
*Matched baseline characteristics*			
Prior BCC radiotherapy, *n*^b^ (%)	5 (7.6%)	(20.6%)	13 (20.6%)
Prior BCC surgery, *n*^b^ (%)	48 (72.7%)	(89.0%)	56 (88.9%)

*Unmatched baseline characteristics *			
Age in years			
Mean	64.6	64.6	61.4
Median	67.0	67.0	62.0
Standard deviation	15.9	15.5	16.9

Age range in years, *n*^b^ (%)			
18–40	6 (9.1%)	(8.6%)	7 (11.1%)
41–64	22 (33.3%)	(31.6%)	26 (41.3%)
≥65	38 (57.6%)	(59.8%)	30 (47.6%)

Race, *n*^b^ (%)			
White	59 (89.4%)	(90.8%)	(100.0%)
Other	7 (10.6%)	(9.2%)	(0.0%)

ECOG status, *n*^b,c^ (%)			
0	44 (66.7%)	(69.3%)	48 (76.2%)
1	16 (24.2%)	(21.5%)	13 (20.6%)
2	4 (6.1%)	(6.0%)	2 (3.2%)

Sex, *n*^b^ (%)			
Male	38 (57.6%)	(60.8%)	35 (55.6%)
Female	28 (42.4%)	(39.2%)	28 (44.4%)

Prior systemic therapy for BCC, *n*^b^ (%)	4 (6.1%)	(5.4%)	7 (11.1%)^d^

BCC: basal cell carcinoma; ECOG: Eastern Cooperative Oncology Group. ^a^BOLT data analysis was based on the 18-month update (i.e., 18 months of patient follow-up) [7]; ERIVANCE summary information was based on the 12-month update (i.e., 21 months of patient follow-up) [16]; ^b^postmatched BOLT results were weighted at the person level; therefore, the number of patients was not available; ^c^two patients had missing ECOG status at baseline; ^d^Systemic or topical.

**Table 5:** Efficacy outcomes parameters.

Efficacy outcome	BOLT^a^, sonidegib 200 mg		ERIVANCE^a^, vismodegib 150 mg (*n* = 63)
	Prematched (*n* = 66)	Postmatched (*n* = 66)	
ORR, *n*^b^ (%) (95% CI^c^)	37 (56.1%) (44.1–68.0)	(56.7%) (44.7–68.6)	30 (47.6%) (35.5–60.6)
Median PFS in months (95% CI)	22.1 (14.8 to NE)	22.1 (14.8 to NE)	9.5 (7.4–14.8)
Median DOR^d^ in months (95% CI)	14.3 (12.0–20.2)	15.7 (12.9–23.1)	NE^e^ (9.0 to NE)
Sensitivity analysis (ERIVANCE-like criteria)			
ORR, % (95% CI)	60.6% (48.4–72.4)	59.5%^c^ (47.6–71.3)	
Median DOR^d^ in months (95% CI)	14.9 (12.0–20.2)	15.7 (12.9–24.0)	

CI: confidence interval; DOR: duration of response; NE: not estimable; ORR: objective response rate; PFS: progression-free survival. ^a^BOLT data analysis was based on the 18-month update (i.e., 18 months of patient follow-up) [7]; ERIVANCE summary information was based on the 12-month update (i.e., 21 months of patient follow-up) [16]; ^b^postmatched BOLT results were weighted at the person level; therefore, the number of patients was not available; ^c^BOLT CIs for ORR were based on Wald asymptotic confidence limits (owing to the incorporation of weights); ^d^DOR was based on investigator review; ^e^median DOR based on independent review facility was reported to be 9.5 months (95% CI: 7.4–21.4).
